# pH-Responsive Polyethylene Oxide-Based Electrospun Nanofibers for Controlled Drug Release in Infected Wound Treatment

**DOI:** 10.3390/polym18020191

**Published:** 2026-01-10

**Authors:** Qian-Yu Yuan, Lan Yang, Bing-Chiuan Shiu, Chien-Teng Hsieh, Ching-Wen Lou, Jia-Horng Lin

**Affiliations:** 1 School of Textile Science and Engineering, Tiangong University, Tianjin 300387, China; 2 Fujian Key Laboratory of Novel Functional Fibers and Materials, Minjiang University, Fuzhou 350108, China; 3 Fujian Engineering Research Center of New Chinese Lacquer Material, College of Material and Chemical Engineering, Minjiang University, Fuzhou 350108, China; 4 Department of Fashion Design and Merchandising, Shih Chien University, Kaohsiung Campus, Kaohsiung City 84550, Taiwan; 5 Department of Bioinformatics and Medical Engineering, Asia University, Taichung City 413305, Taiwan; 6 Advanced Medical Care and Protection Technology Research Center, Department of Fiber and Composite Materials, Feng Chia University, Taichung City 407102, Taiwan; 7 School of Chinese Medicine, China Medical University, Taichung City 404333, Taiwan

**Keywords:** polyethylene oxide, chitosan, drug delivery, composite fiber membranes

## Abstract

Infected wounds form a complex microenvironment that creates difficulties for drug delivery. In this study, a composite fiber membrane based on polyethylene oxide (PEO) was prepared. The intention was to achieve on-demand drug release and integrate multiple functions by adjusting the material composition. The membrane uses PEO as the main framework and contains chitosan (CS) and ascorbic acid (Asc). CS leads to an increase in fiber diameter, while Asc makes the fibers thinner. The two components act together to influence the microstructure. In vitro drug release experiments showed that changing the CS content in the PEO matrix can affect the initial release rate and the duration of sustained release. The membrane also shows sensitivity to pH. Under slightly acidic conditions, drug release becomes faster, which is similar to the state of infected wounds. In addition, the membrane maintains antioxidant activity and can inhibit *Escherichia coli* (*E. coli*) and *Staphylococcus aureus* (*S. aureus*). These results suggest that PEO-based composite fibers may be useful in drug delivery and tissue repair.

## 1. Introduction

The skin is the main defense barrier that maintains the body’s homeostasis [1,2,3]. Once the epidermal structure is damaged, the resulting wound not only becomes a channel for microbial invasion but also interferes with the normal healing process [4,5]. Wound repair typically follows four interrelated biological stages: hemostasis, inflammation, proliferation and remodeling [6]. Due to the complexity of this process, complications such as bacterial infection, abnormal scarring or recurrent bleeding often occur at the wound site [7,8]. Therefore, the use of functional dressings has become a key strategy for promoting healing.

Electrospinning technology has demonstrated unique value in the field of medical dressings [9,10]. The nanofiber materials manufactured through this technology have a special microstructure that can effectively prevent bacterial penetration without interfering with wound metabolism. Its high porosity structure not only facilitates the penetration of water and air but also promotes tissue regeneration by simulating the extracellular matrix (ECM) environment [11]. In addition, fibrous scaffolds can simulate the microenvironment required for human cell growth and promote the directional migration of repair cells [12]. With the development of nanotechnology, drug controlled release has gradually become an important direction in the design of wound dressings [13,14]. The ideal wound dressing should be capable of providing continuous and controllable drug release to adapt to different stages of healing, thereby optimizing the therapeutic effect of the drug [15], while maintaining adequate biocompatibility, including hemocompatibility, for safe wound contact [16]. Meanwhile, wound dressings with controllable drug release functions can dynamically regulate the microenvironment of the wound surface, effectively inhibit infection, alleviate inflammatory responses, and promote cell proliferation and tissue repair [17]. This method not only enhances the therapeutic effect by improving the efficiency of drug utilization, but also significantly reduces the toxic and side effects caused by the initial sudden release and long-term accumulation of drugs [18,19], ensuring that the repair dressing can perform optimally at each critical stage of healing.

Polyethylene oxide (PEO) is a polymer with crystalline, thermoplastic, biocompatible and biodegradable properties, featuring excellent thermal stability, non-toxicity and spinnability [20,21,22,23]. Due to its high hydrophilicity and biocompatibility, PEO has been widely applied in many fields, especially in the preparation of composite fiber membranes (NFM) [24,25,26]. PEO can combine with other polymers such as chitosan (CS) through hydrogen bonds, reducing the viscosity, surface tension and electrical conductivity of the solution, thereby helping to form a dense nanofiber network and significantly improving the spinnability of nanofibers [27,28]. Chitosan (CS), as a natural high-molecular basic polysaccharide, exhibits excellent biocompatibility and biodegradability after deacetylation treatment [29,30,31,32]. Because chitosan chains carry a positive charge, they can bind to proteins and promote cell adhesion. In addition, the oligosaccharide structure in chitosan can also stimulate macrophages and chemically attract neutrophils [33,34,35]. Although chitosan has these biological functions, its rigid molecular chain structure and polycationic characteristics make pure chitosan difficult to dissolve and spin into composite fiber membranes. Therefore, by blending chitosan with PEO and preparing composite fiber membranes through electrospinning [36,37], not only can the spinnability of chitosan be improved, but also the mechanical properties and biocompatibility of the composite fiber membranes can be significantly enhanced, further strengthening the functionality of the drug delivery system. Although electrospun PEO/chitosan composite systems have been widely reported for biomedical applications, most previous studies primarily focused on improving the spinnability of chitosan or enhancing antibacterial performance, with limited attention paid to the role of PEO-dominated matrices in regulating drug release behavior under wound-relevant conditions. In the present study, we emphasize a composition-driven design strategy in which PEO serves as the primary structural and release-controlling component, while chitosan and ascorbic acid play complementary roles in microstructure modulation and biological functionality. By systematically adjusting the CS content under fixed processing conditions, we establish a clear relationship between fiber microstructure, pH-responsive drug release behavior, and release kinetics relevant to infected wound microenvironments. This work therefore provides a focused and mechanistic understanding of PEO-based electrospun fiber membranes as controllable drug delivery platforms for wound treatment.

Ascorbic acid (Asc), with its powerful antioxidant activity, can reduce oxidative damage to cells, promote collagen synthesis, and thereby accelerate wound healing [38,39]. Ascorbic acid plays a relatively important role in the process of wound healing by promoting the hydroxylation of proline and lysine residues and enhancing the stability of collagen [40,41]. Therefore, incorporating ascorbic acid as a drug component into the composite fiber membrane can inhibit bacterial infection, reduce inflammatory response, and thereby accelerate tissue repair [42]. However, ascorbic acid is susceptible to environmental influences in practical applications, such as temperature, light and oxidation, which leads to stability issues [43,44,45]. Therefore, by using nanofiber carriers to encapsulate ascorbic acid, not only can its stability be enhanced, but also the controllable release of the drug can be achieved.

Therefore, this paper aims to develop a PEO/CS composite fiber membrane loaded with ascorbic acid for wound healing applications. The performance of PEO/CS composite fiber membranes loaded with ascorbic acid in terms of structure and antibacterial properties was evaluated by multiple characterization methods such as in vitro release and antibacterial performance tests. Through the optimization of the technology, composite fiber membranes with ideal degradation rates and controllable drug release characteristics were prepared, demonstrating the potential of PEO as a drug controlled-release carrier. It provides a new treatment option for wound healing.

## 2. Materials and Methods

### 2.1. Materials

Chitosan (CS, 200–400 mPa·s; DD, 75–85%), polyethylene oxide (PEO, average Mv 3,000,000), and ascorbic acid (Asc) were purchased from Aladdin (Shanghai, China). Agar powder (gel strength: 1400), peptone (BR, 01-001), and yeast extract (BR) were obtained from Combest Biotechnology Co., Ltd. (Beijing, China), Beijing Aobor Biotechnology Co., Ltd. (Beijing, China), and Maclin Biochemical Co., Ltd. (Shanghai, China). Distilled water was prepared in the laboratory, and two bacterial strains, *S. aureus* (ATCC 6538) and *E. coli* (ATCC 8739), were cultured. Sodium chloride (AR) and glacial acetic acid (AR) were sourced from China National Pharmaceutical Group Chemical Reagent Co., Ltd. (Shanghai, China). Phosphate-buffered saline (PBS, pH 6.8–8) was obtained from Beijing Taiyang Biotechnology Co., Ltd. (Beijing, China).

### 2.2. Preparation of PEO/CS/Asc Composite Fiber Membranes

To prepare the electrospinning solution of PEO/CS/Asc, a 70 wt% acetic acid solution was first prepared. Subsequently, 4 wt% PEO and 1–4 wt% CS were separately dissolved in 60 wt% acetic acid solution under vigorous stirring at room temperature until uniform and transparent mixtures were formed (C/P-1%; C/P-2%; C/P-3%; C/P-4%). The solutions were mixed in predetermined proportions, and then a fixed concentration of 1 wt% Asc was added. The mixed solution was then subjected to ultrasonic treatment to ensure uniform dispersion, resulting in four different CS/PEO/Asc solutions (A@C/P-1%; A@C/P-2%; A@C/P-3%; A@C/P-4%).

During the electrospinning process, the prepared solution is filled into a 10 mL plastic syringe. The flow rate is maintained at 0.8 mL/h. Apply a high-voltage electric field (23 kV) at a fixed distance of 15 cm between the needle tip and the cylindrical collector. Nanofibers are uniformly deposited on a rotating mandrel collector (400 rpm). The obtained electrospun composite fiber film was vacuum-dried to remove the residual solvent.

### 2.3. Characteristics

#### 2.3.1. Field Emission Scanning Electron Microscopy (SEM)

The morphology of electrospun fibers was examined using a field emission scanning electron microscope (Hitachi SU8010, Tokyo, Japan). All the samples were gold-sprayed before imaging. The quantitative analysis of fiber diameters was accomplished with the aid of ImageJ v1.53, and approximately 100 fibers were randomly counted for each image. Based on these measurement data, the corresponding diameter distribution histogram was generated through the Origin 2021.

#### 2.3.2. Fourier Transform Infrared Spectroscopy (FTIR)

The chemical properties of the prepared nanofiber structures were analyzed using a Fourier Transform infrared (FTIR) spectrometer (NICOLET IS5, Thermo Fisher Scientific Co., Ltd., Shanghai, China), and the recorded spectral range was 500–4000 cm^−1^.

#### 2.3.3. Contact Angle

The water contact angle was measured using a dynamic contact angle analyzer (DSA30S, KRÜSS GmbH, Hamburg, Germany). The samples were cut into rectangles and dried to ensure a dry surface. Three parallel tests were conducted, and the average value was reported as the final measurement.

#### 2.3.4. In Vitro Release

The composite fiber membranes were cut into specified sizes and immersed in PBS of different pH values. The containers were sealed and placed in a constant temperature shaker at 37 °C to simulate drug release. At predetermined time points (1, 2, 4, 6, 8, 10, and 24 h), a certain amount of release medium was taken out and replaced with an equal volume of fresh PBS. The maximum absorption wavelength of the nanomaterials was tested using a UV 2600 UV-Vis spectrophotometer. The values were then substituted into the corresponding standard calibration curve equation to calculate the cumulative drug release rate. The formula is as follows:

$$Q\;(\%)\;=\;\frac{C_{n}V\;+\;\sum_{i=1}^{n-1}C_{i}V_{i}}{m_{0}}\;\times \;100\%$$

#### 2.3.5. Antibacterial Activity

The antibacterial activity of the composite fiber membrane against *E. coli* and *S. aureus* was evaluated by the colony counting method. Firstly, the two strains were separately cultured in LB liquid medium for 24 h under shaking conditions (37 ± 1 °C, 110 rpm). Subsequently, the cultures were serially diluted with sterile PBS buffer (pH = 7.4) until the bacterial suspension concentration reached 3 × 10^5^ to 4 × 10^5^ CFU/mL, which was used as the standardized inoculum.

Antibacterial tests were conducted in accordance with the GB/T 20944.3-2008 standard (Textiles—Evaluation for antibacterial activity—Part 3: Shake flask method. Standardization Administration of China: Beijing, China, 2008). The samples were mixed with the above-mentioned standardized bacterial suspension in PBS and incubated in a constant temperature shaker at 24 ± 1 °C and 150 rpm for 18 h. After incubation, the surviving bacteria were quantified by the plate spread method. 200 μL of the diluted solution was evenly spread on LB agar plates. All plates were inverted and cultured at 37 ± 1 °C for 24 h, and then the formed colonies were counted. Three parallel samples were set for all experiments, and the antibacterial rate (BR%) was calculated according to the following formula.

$$\mathrm{BR}\%=\frac{B-A}{B}\;\times \;100\%$$

Among them, A and B, respectively, represent the colony count values of the experimental group and the control group.

#### 2.3.6. Antioxidant Activity

The antioxidant activity of the composite fiber membranes was evaluated by the DPPH method. A certain mass of the composite fiber membrane samples was accurately weighed and immersed in 5 mL of freshly prepared 0.1 mM DPPH ethanol solution. Both the solution preparation and the reaction process were carried out in the dark. At the preset time points (5, 10, 20, 30, 40, 50, 60 and 120 min), 3 mL of the solution was taken for detection, and an equal volume of solvent was added to maintain a constant total volume. The analysis was conducted using a UV 2600 ultraviolet-visible spectrophotometer. Each sample was measured three times, and the DPPH radical scavenging rate was ultimately calculated according to the following formula.

$$\mathrm{DPPH}\;\mathrm{radical}\;\mathrm{scavenging}\;\mathrm{percentage}\;(\%)=\;\frac{A_{0\;}-{\;A}_{1}}{A_{0}}\;\times \;100\%$$

## 3. Results

### 3.1. Microscopic Morphology of Nanofibers

**Figure 1:**
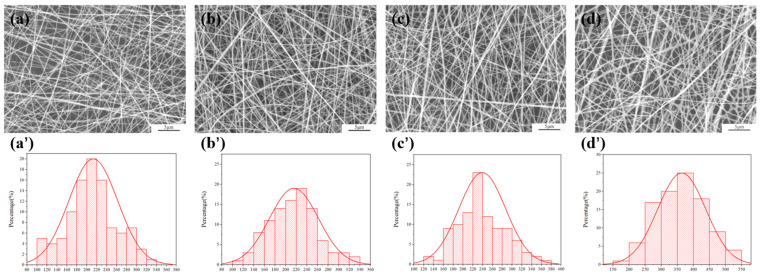
SEM observation figure and fiber distribution of nanometer fiber membrane (**a**,**a’**) C/P-1%, (**b**,**b’**) C/P-2%, (**c**,**c’**) C/P-3%, (**d**,**d’**) C/P-4%.

The composite fiber membrane provides the necessary physical support for cells, promoting their adhesion, proliferation, migration and differentiation. Meanwhile, these composite fiber membranes have smaller pores and a higher surface area to volume ratio, which is conducive to subsequent drug release behavior and cell interactions. As shown in Figure 1, with the increase in CS content, the average diameter of nanofibers shows a significant increasing trend, increasing from 210 ± 23 nm to 359 ± 21 nm, respectively. The increase in the diameter of this fiber is mainly attributed to CS, as a macromolecular polyelectrolyte, which raises the concentration of polymer chains and the degree of entanglement between molecules in the solution, leading to an increase in CS content and a significant rise in solution viscosity. During the electrospinning process, a higher solution viscosity will restrict the stretching of the polymer jet, thereby increasing the fiber diameter.

**Figure 2:**
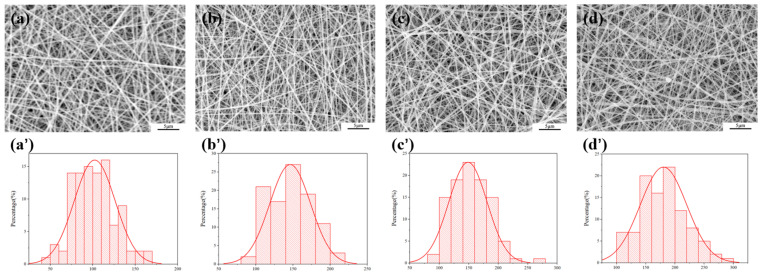
SEM observation figure and fiber distribution of nanometer fiber membrane (**a**,**a’**) A@C/P-1%, (**b**,**b’**) A@C/P-2%, (**c**,**c’**) A@C/P-3%, (**d**,**d’**) A@C/P-4%.

Subsequently, Asc was added as a drug to the C/P mixture. As shown in Figure 2, the addition of Asc significantly refined the fiber morphology. Under all C/P ratios, the fiber diameters decreased significantly after the addition of Asc, with an overall distribution ranging from 102 to 180 nm. Moreover, the fiber morphology became more uniform, with a reduction in bead-like structures and adhesion. As a small molecule organic acid, Asc may interfere with the hydrogen bond network between polymer chains through its polar groups, reducing the solution viscosity. Additionally, the addition of Asc also increased the solution conductivity, thereby enhancing the stretching effect of the electric field on the jet. This forms a competitive relationship with the fiber thickening effect of CS. Therefore, precisely regulating the ratio of the two can effectively and directionally adjust the final microstructure of the nanofibers.

### 3.2. Chemical Structure and Surface Property Analysis

**Figure 3:**
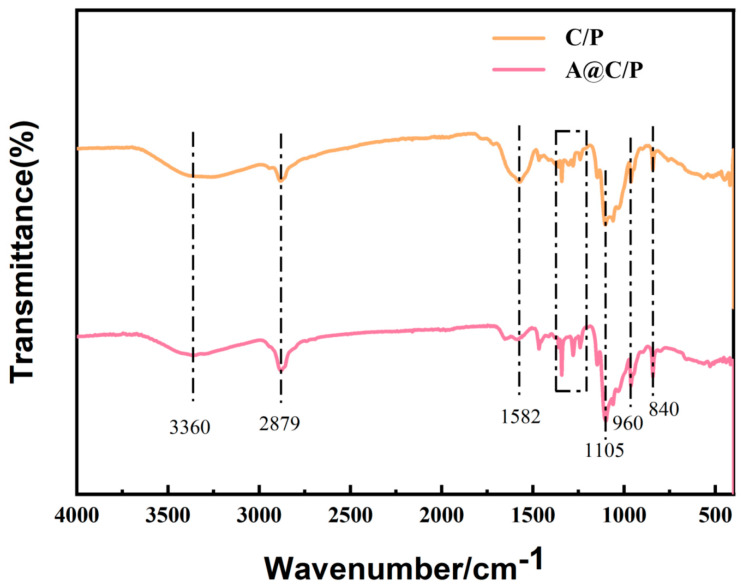
Infrared analysis of C/P fiber membranes and A@C/P drug-loaded membranes.

Figure 3 shows the FT-IR spectra of the C/P fiber membranes and the A@C/P drug-loaded membranes. By comparing the two spectra, it can be observed that there is a broad and strong absorption peak at around 3360 cm^−1^ in the C/P fiber membranes, which is mainly attributed to the stretching vibration of O-H and N-H groups in the CS molecule, and remains significant in the A@C/P drug-loaded membranes. Both spectra show a distinct absorption peak at 2879 cm^−1^, which is typically attributed to the stretching vibration of the C-H group in the -CH_2_- of PEO and CS. In the C/P fiber membranes, the absorption peak at 1582 cm^−1^ is only weak; however, in the A@C/P drug-loaded membranes, the absorption peak at 1582 cm^−1^ is significantly enhanced and becomes sharper, which is mainly attributed to the stretching vibration of the carbonyl (C=O) group in the Asc molecule, strongly confirming that Asc has been successfully encapsulated in the nanofiber system. Additionally, both spectra exhibit a strong absorption peak at 1105 cm^−1^, which is typically attributed to the stretching vibration of the C-O-C ether bond in PEO and the vibration of the C-O alcohol group in CS and Asc. The peaks near 960 cm^−1^ and 840 cm^−1^ are related to the molecular chain vibration of PEO and the out-of-plane bending vibration of C-H, respectively.

**Figure 4:**
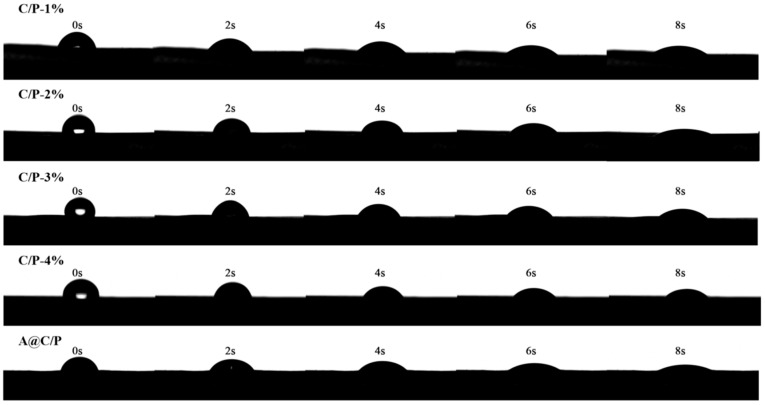
Water contact Angle test of C/P fiber membranes and A@C/P drug-loaded membranes.

Hydrophilic surfaces can provide a favorable platform for cell adhesion and proliferation. As shown in Figure 4, the prepared composite fiber membranes exhibit excellent hydrophilic properties. When water droplets are dropped onto the surfaces of all the composite fiber membranes with different CS contents, they are completely absorbed and spread within a short time. However, as the CS content increases, the time required for the water droplets to be completely absorbed by the composite fiber membranes slightly extends. C/P-1% can fully absorb water droplets within about 1–2 s, whereas C/P-4% may require nearly 4–5 s for complete absorption. Although CS itself is rich in polar groups, its semi-rigid molecular chains and strong inter-chain hydrogen bonding make it play the role of a structural reinforcing skeleton in the fiber network. When the CS content is low, PEO dominates in the matrix, and the numerous ether oxygen atoms on the PEO molecular chains can form hydrogen bonds with water molecules. Upon contact with water, the PEO segments rapidly swell and dissolve, causing the fiber structure to disintegrate quickly and absorb water at an extremely fast rate. However, when the CS content increases, the formed CS network becomes denser and more robust, which can better maintain the fiber morphology while absorbing water. Therefore, a higher CS content not only provides hydrophilicity but also slows down the degradation rate of the matrix, which is consistent with the conclusion of in vitro drug release. The composite fiber membranes loaded with Asc absorb water even faster because Asc is a small molecule, highly polar compound rich in multiple hydroxyl groups. When it is encapsulated in the fibers, especially distributed on the fiber surface, it can act as an “anchor point” for water molecule adsorption, greatly accelerating the initial spreading process of water droplets on the fiber surface and thus absorbing water more quickly.

### 3.3. Analysis of In Vitro Drug Release Behavior

**Figure 5:**
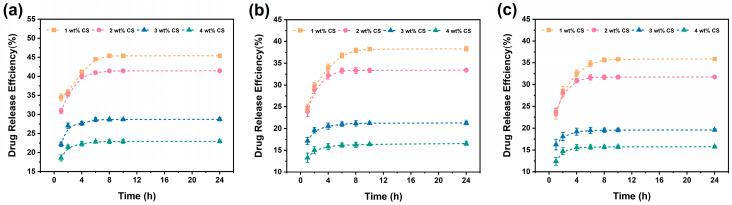
In vitro release of A@C/P drug-loaded membranes at different pH values (**a**) pH 6.8 (**b**) pH 7.4 (**c**) pH 8.

As shown in Figure 5, the content of CS is the core factor controlling the drug release rate. Under the same pH conditions, as the CS content increased from 1 wt% to 4 wt%, the drug release rate slowed down significantly. When the pH was 6.8, within 2 h, A@C/P-1% released nearly 30% of the drug, showing a relatively fast initial release, while the release rate of the A@C/P-4% dropped to approximately 15% at the same time. And this trend persisted for 24 h, which also proved that increasing the CS content could achieve sustained drug release. Increasing the CS content could effectively inhibit and regulate the initial burst release degree. Because the PEO molecule contains a large number of ether oxygen atoms, it can form hydrogen bonds with water molecules, enabling it to rapidly swell and dissolve in aqueous solutions.

However, the solubility of CS is relatively low, and its dissolution rate is affected by pH. Therefore, in the early stage of the release of the composite fiber membrane, the rapid dissolution of PEO is dominant, which leads to the rapid release of the near-surface drug, resulting in a certain burst release. Subsequently, as PEO gradually dissolved, CS formed a relatively stable and cross-linked network skeleton at neutral pH. As a physical barrier for drug diffusion, the density and strength of the CS skeleton directly affect the diffusion rate of drug molecules. When the CS content is low, the CS skeleton left after the dissolution of PEO is relatively loose, and its hindrance to drug diffusion is limited, resulting in a faster drug release. When the content of CS is relatively high, the CS skeleton formed after the dissolution of PEO is denser and tougher, significantly increasing the diffusion path and resistance for drug molecules to penetrate this network, thereby effectively delaying the continuous release of the drug. Through the synergistic effect of the rapid dissolution of PEO and the stable framework of CS, the drug release rate can be effectively controlled by adjusting the content of CS. With increasing CS content, a denser polymer network is formed within the composite fibers, which becomes the dominant factor governing drug diffusion. Although protonation of amino groups under mildly acidic conditions can still induce network swelling, its relative contribution to accelerating drug release is reduced. As a result, the apparent pH dependence of the release behavior becomes less pronounced at higher CS contents.

Meanwhile, the drug release of the composite fiber membrane also shows a distinct pH responsiveness. By comparing the release curves of the samples in PBS buffer solutions with different pH values, it can be found that the drug release rate of the composite fiber membrane is significantly faster in a slightly acidic environment than in neutral or slightly alkaline environments. For instance, taking A@C/P-2% as an example, its cumulative release amount at 24 h is 41% at pH 6.8, 33% at pH 7.4, and 31% at pH 8.0. The pKa value of the amino group of CS is approximately 6.5. At pH 6.8, which is a slightly acidic environment, it is close to its pKa value, and some amino groups will be protonated to form positively charged -NH_3_^+^ groups. These charged groups generate electrostatic repulsion, causing the CS polymer chain segments to stretch, leading to swelling of the entire fiber network and an increase in pore size, which facilitates the faster diffusion of the encapsulated drug molecules from the interior of the fibers. In contrast, at pH 7.4 and pH 8.0 in neutral or slightly alkaline environments, the amino groups of CS mainly exist in the non-protonated -NH_2_ form, and the inter-chain hydrogen bonding interaction is enhanced, resulting in a relatively compact network structure that exerts a stronger hindrance to drug diffusion, thus the release rate is slower. For clarity, the key differences in drug release behavior among samples with different CS contents are summarized in Table 1.

**Figure 6:**
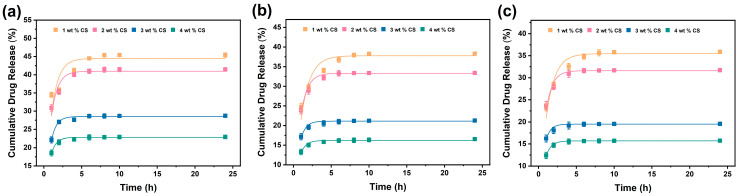
First-order kinetic fitting of in vitro drug release profiles of A@C/P drug-loaded membranes under different pH conditions: (**a**) pH 6.8, (**b**) pH 7.4, and (**c**) pH 8.0.

To deeply explore the release mechanism of drug molecules from composite fiber membranes under different pH environments and CS contents, we fitted the in vitro release data with kinetic models, including zero-order kinetics, first-order kinetics, Higuchi model and Ritger–Peppas model. The coefficient of determination (*R*^2^) of the fitting results was used as the key indicator to evaluate the consistency between the model and the experimental data. The details are shown in Figure 6 and Figures S1–S3, and Table 2, Table 3 and Table 4.

According to the table data, under all tested pH conditions (pH 6.8, 7.4, 8.0) and CS concentrations (1–4 wt%), the first-order kinetic equation had a significantly better fit than other models. In most cases, the *R*^2^ value of the first-order kinetic model was the highest among all models and generally exceeded 0.8, demonstrating consistency with the experimental data.

The first-order kinetic model usually refers to a process where the drug release rate is directly proportional to the remaining drug concentration in the matrix. The drug release behavior is mainly a diffusion process driven by the concentration gradient. For the PEO/CS/Asc composite fiber membrane in this study, this mechanism is in line with its physicochemical properties. The matrix of this composite fiber membrane is composed of hydrophilic PEO and CS. When it comes into contact with the release medium, the fibers will rapidly absorb water and swell. The drug molecules (Asc) are encapsulated in the porous network formed by the swollen fibers, and their release to the external medium mainly depends on the diffusion within the network pores.

Furthermore, through the fitting of the Ritger–Peppas model, the type of diffusion can be further analyzed. It can be seen from the table that the release exponent n values under all conditions are far less than 0.45. For the film dosage form, this indicates that the drug release mechanism is Fickian diffusion, that is, diffusion is the dominant process. This is also consistent with the diffusion-controlled mechanism revealed by the fitting results of the first-order kinetic model.

### 3.4. Evaluation of the Biological Properties of Materials

**Figure 7:**
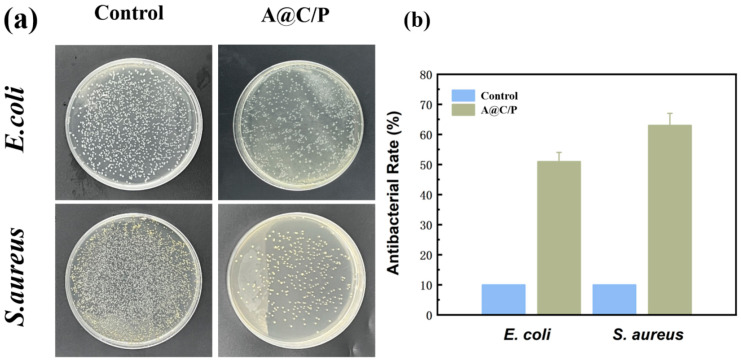
(**a**) colony status (**b**) sterilization rate test of the A@C/P drug-loaded membranes co-cultured with *E. coli* and *S. aureus* for 24 h.

To assess the potential of the composite fiber membrane in preventing or treating wound infections, we used the colony counting method to detect the inhibitory effects on Gram-negative *E. coli* and Gram-positive *S. aureus*. As shown in Figure 7, compared with the control group, the A@C/P drug-loaded membranes could effectively reduce the number of bacterial colonies, demonstrating a certain inhibitory effect. PEO itself mainly serves as a fibrous matrix core carrier and does not have antibacterial activity, so the antibacterial activity is mainly attributed to the synergistic effect of CS and Asc. CS, as a key antibacterial agent, has amino groups on its molecular chain that protonate in an aqueous environment to form positively charged cationic groups. These cations can firmly bind to the negatively charged bacterial cell membrane/wall surface through electrostatic attraction. This binding disrupts the integrity of the cell membrane, increases its permeability, and leads to the leakage of important intracellular substances, ultimately causing bacterial death. Asc itself has certain antibacterial capabilities. It can interfere with the normal metabolism of bacteria by affecting the intracellular redox balance or generating a certain amount of ROS under specific conditions. Additionally, the weak acidity of Asc may help maintain a local microenvironment conducive to the protonation of CS amino groups, thereby enhancing its cationic antibacterial activity and generating a synergistic effect with CS.

**Figure 8:**
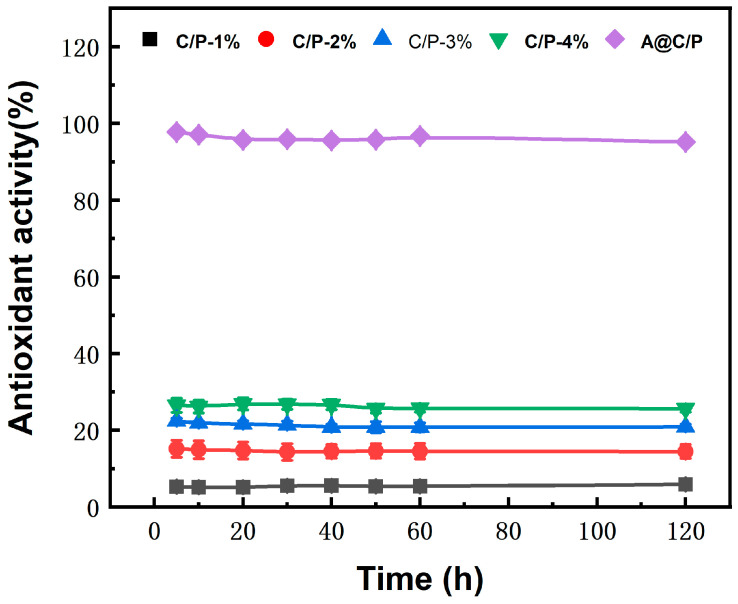
Antioxidant properties of C/P fiber membranes with different CS contents and A@C/P drug-loaded membranes.

Oxidative stress is often present during the wound healing process, and excessive free radicals can delay the healing process. In this study, the antioxidant capacity of A@C/P drug-loaded membranes was evaluated by the DPPH method. As shown in Figure 8, the C/P fiber membranes exhibit relatively low free radical scavenging activity, indicating that PEO and CS possess only limited intrinsic DPPH radical scavenging ability. With increasing CS content, the DPPH radical scavenging efficiency showed a modest increase, although the overall antioxidant capacity remained low compared to Asc-loaded samples. This behavior is consistent with previous reports, where chitosan exhibits weak but measurable antioxidant activity associated with its hydroxyl and amino groups.

However, when Asc is loaded into the composite fiber membrane, the A@C/P drug-loaded membranes demonstrates a powerful and rapid free radical scavenging ability. Within 5 min of contact with DPPH solution, its antioxidant activity rapidly reached a high level of 97%, and it remained above 91% for the following 2 h. This performance is attributed to the enediol structure on the Asc molecule, which can efficiently provide electrons or hydrogen atoms to directly eliminate free radicals. The C/P fiber membranes matrix, as a stable carrier, effectively encapsulates and continuously releases highly active Asc through its porous nanostructure. This ensures that the composite fiber membrane can provide immediate and long-lasting antioxidant protection for wounds, thereby facilitating healing.

## 4. Discussion

In this study, a composite fiber membrane based on A@C/P drug-loaded membranes was prepared and characterized, which can be used as a potential multifunctional wound dressing. The characteristics of the composite fiber membrane in terms of microstructure regulation, drug release behavior and biological functions were emphatically explored. At the material structure level, the introduction of CS significantly increased the diameter of nanofibers, with the average fiber diameter increasing from approximately 210 nm to about 359 nm, while the addition of Asc effectively refined the fibers and improved their morphological uniformity, reducing the fiber diameter to the range of approximately 102–180 nm, demonstrating the synergistic effect of the two components on the microstructure of the fibers. Meanwhile, this composite fiber membrane has good hydrophilicity, which helps it form an effective interface with wound fluid.

The in vitro drug release study revealed that the release characteristics of A@C/P drug-loaded membranes are jointly affected by matrix dissolution and environmental pH value. By adjusting the content of CS, the initial release rate of the drug and the overall sustained-release cycle can be effectively affected. Specifically, an increase in CS content can slow down the release and inhibit the rapid release in the initial stage. Under the same pH conditions, increasing CS content consistently slowed down drug release and reduced the early-stage burst effect. For instance, at pH 6.8, A@C/P-1% released nearly 30% within 2 h, whereas A@C/P-4% released approximately 15% at the same time point, indicating that higher CS content effectively suppresses the initial rapid release.

In addition, the composite fiber membrane exhibits a faster drug release rate in a slightly acidic environment, which is consistent with the pH responsiveness of the CS amino group. This pH-responsive trend is reflected by the higher cumulative release at pH 6.8 compared with pH 7.4 and pH 8.0; for example, A@C/P-2% reached 41% at pH 6.8 after 24 h, versus 33% at pH 7.4 and 31% at pH 8.0. The acceleration of release under mildly acidic conditions was observed for all compositions, while the extent of pH-induced enhancement was modulated by CS content due to the structural restriction imposed by the denser CS network. Moreover, the fiber composite fiber membrane also exhibits antibacterial ability and highly efficient antioxidant activity brought about by the synergy of CS and Asc. In conclusion, through ingenious component design, the PEO-based composite fiber membrane has been successfully endowed with the ability to achieve controllable drug release and multifunctional protection, providing a promising new strategy for the management of infected wounds.

## Figures and Tables

**Table 1:** Summary of the drug release behaviors of A@C/P electrospun fiber membranes with varying chitosan contents.

Sample	Initial Burst Release	Overall Release Level (24 h)	Main Effect of Increasing CS Content	pH-Dependent Release Trend
A@C/P-1%	Pronounced	High	Lower diffusion barrier	Most pronounced pH dependence (faster at pH 6.8)
A@C/P-2%	Moderate	Medium–high	Increased diffusion resistance	Clear pH dependence (faster at pH 6.8)
A@C/P-3%	Suppressed	Medium	Denser CS network limits diffusion	Less pronounced pH dependence
A@C/P-4%	Minimal	Low	Highly restricted diffusion dominated by compact matrix	Weakest pH dependence among all samples

**Table 2:** Kinetic model (pH 6.8).

Sample	Kinetic Model	Formula	*R* ^2^
A@C/P-1%	Zero order	*Q* = 39.4 + 0.4*t*	0.3950
	First order	*Q* = 44.5 (1 − e^−1.08^*^t^*)	0.7024
	Higuchi	*Q* = 3.1*t*^1/2^ + 34.6	0.6209
	Ritger–Peppas	*Q* = 35.6*t*^0.1^	0.7933
A@C/P-2%	Zero order	*Q* = 37.8 + 0.2*t*	0.2869
	First order	*Q* = 41 (1 − e^−1.21*t*^)	0.8947
	Higuchi	*Q* = 1.6*t*^1/2^ + 35.2	0.449
	Ritger–Peppas	*Q* = 35.1*t*^0.07^	0.65
A@C/P-3%	Zero order	*Q* = 27 + 0.1*t*	0.2420
	First order	*Q* = 28.5 (1 − e^−1.48*t*^)	0.9528
	Higuchi	*Q* = 0.8*t*^1/2^ + 25.8	0.3796
	Ritger–Peppas	*Q* = 25.6*t*^0.04^	0.5614
A@C/P-4%	Zero order	*Q* = 21.9 + 0.1*t*	0.2100
	First order	*Q* = 22.8 (1 − e^−1.63*t*^)	0.9458
	Higuchi	*Q* = 0.5*t*^1/2^ + 21	0.3709
	Ritger–Peppas	*Q* = 20.7*t*^0.04^	0.5956

**Table 3:** Kinetic model (pH 7.4).

Sample	Kinetic Model	Formula	*R* ^2^
A@C/P-1%	Zero order	*Q* = 33.2 + 0.3*t*	0.3647
	First order	*Q* = 37.8 (1 − e^−0.84*t*^)	0.8881
	Higuchi	*Q* = 2.2*t*^1/2^ + 29.3	0.5477
	Ritger–Peppas	*Q* = 29.4*t*^0.1^	0.7329
A@C/P-2%	Zero order	*Q* = 31.9 + 0.1*t*	0.1363
	First order	*Q* = 33.3 (1 − e^−1.16*t*^)	0.9635
	Higuchi	*Q* = 0.9*t*^1/2^ + 30.2	0.2734
	Ritger–Peppas	*Q* = 29.3*t*^0.05^	0.4958
A@C/P-3%	Zero order	*Q* = 20.1 + 0.1*t*	0.2734
	First order	*Q* = 21.1 (1 − e^−1.56*t*^)	0.9233
	Higuchi	*Q* = 0.6^1/2^ + 19.1	0.4576
	Ritger–Peppas	*Q* = 18.9*t*^0.05^	0.6808
A@C/P-4%	Zero order	*Q* = 15.2 + 0.1*t*	0.4013
	First order	*Q* = 16.2 (1 − e^−1.59*t*^)	0.8645
	Higuchi	*Q* = 0.5*t*^1/2^ + 14.5	0.561
	Ritger–Peppas	*Q* = 14.5*t*^0.05^	0.7505

**Table 4:** Kinetic model (pH 8).

Sample	Kinetic Model	Formula	*R* ^2^
A@C/P-1%	Zero order	*Q* = 30.4 + 0.3*t*	0.4174
	First order	*Q* = 35.4 (1 − e^−0.88*t*^)	0.9357
	Higuchi	*Q* = 2.2*t*^1/2^ + 26.9	0.5979
	Ritger–Peppas	*Q* = 27.4*t*^0.1^	0.7666
A@C/P-2%	Zero order	*Q* = 29.7 + 0.1*t*	0.4174
	First order	*Q* = 31.6 (1 − e^−1.23*t*^)	0.9471
	Higuchi	*Q* = 1.1*t*^1/2^ + 27.8	0.4005
	Ritger–Peppas	*Q* = 27.5*t*^0.06^	0.6152
A@C/P-3%	Zero order	*Q* = 18.9 + 0.1*t*	0.189
	First order	*Q* = 19.5 (1 − e^−1.69*t*^)	0.9207
	Higuchi	*Q* = 0.4*t*^1/2^ + 18.2	0.3389
	Ritger–Peppas	*Q* = 17.9*t*^0.03^	0.5714
A@C/P-4%	Zero order	*Q* = 14.9 + 0.1*t*	0.2265
	First order	*Q* = 15.7 (1 − e^−1.51*t*^)	0.9868
	Higuchi	*Q* = 0.4*t*^1/2^ + 14.3	0.3569
	Ritger–Peppas	*Q* = 14.2*t*^0.04^	0.5463

## Data Availability

The data that support the findings of this study are available on request from the corresponding author. The data are not publicly available due to privacy or ethical restrictions.

## Supplementary Materials

The following supporting information can be downloaded at: https://www.mdpi.com/article/10.3390/polym18020191/s1, Figure S1: Zero-order kinetic fitting of in vitro drug release profiles of A@C/P drug-loaded membranes with different CS contents under different pH conditions: (a) pH 6.8, (b) pH 7.4, and (c) pH 8.0; Figure S2: Higuchi model fitting of in vitro drug release profiles of A@C/P drug-loaded membranes with different CS contents under different pH conditions: (a) pH 6.8, (b) pH 7.4, and (c) pH 8.0; Figure S3: Ritger–Peppas model fitting of in vitro drug release profiles of A@C/P drug-loaded membranes with different CS contents under different pH conditions: (a) pH 6.8, (b) pH 7.4, and (c) pH 8.0.
