# Supplementary material for: pH-Responsive Polyethylene Oxide-Based Electrospun Nanofibers for Controlled Drug Release in Infected Wound Treatment
Source: Polymers (Basel). 2026 Jan 10;18(2):191. doi: 10.3390/polym18020191 (PMC12846034; doi:10.3390/polym18020191)
Supplement: Supplementary file 1 [file polymers-18-00191-s001.zip › polymers-4070546-supplementary.pdf]

pH-Responsive Polyethylene Oxide-Based Electrospun Nanofibers for Controlled Drug Release in Infected Wound Treatment

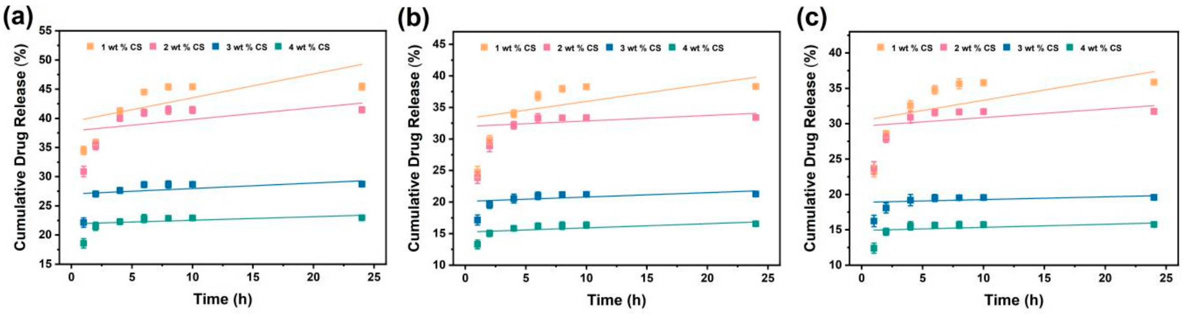

Figure S1. Zero-order kinetic fitting of in vitro drug release profiles of A@C/P drug-loaded membranes with different CS contents under different pH conditions: (a) pH 6.8, (b) pH 7.4, and (c) pH 8.0.

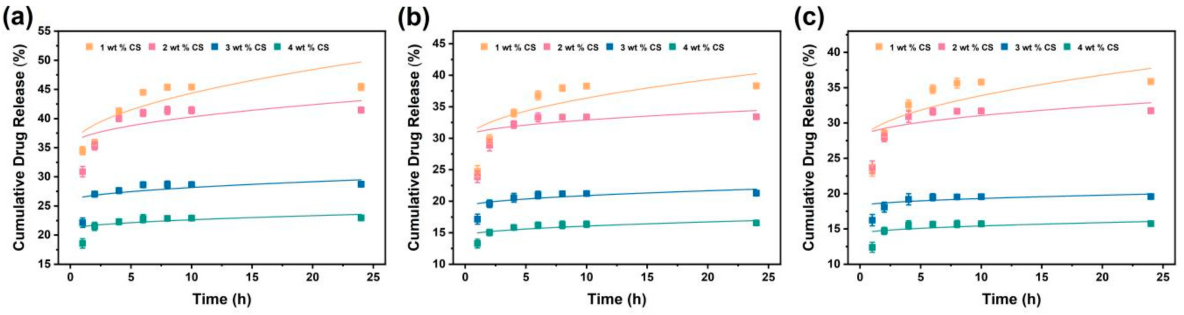

Figure S2. Higuchi model fitting of in vitro drug release profiles of A@C/P drug-loaded membranes with different CS contents under different pH conditions: (a) pH 6.8, (b) pH 7.4, and (c) pH 8.0.

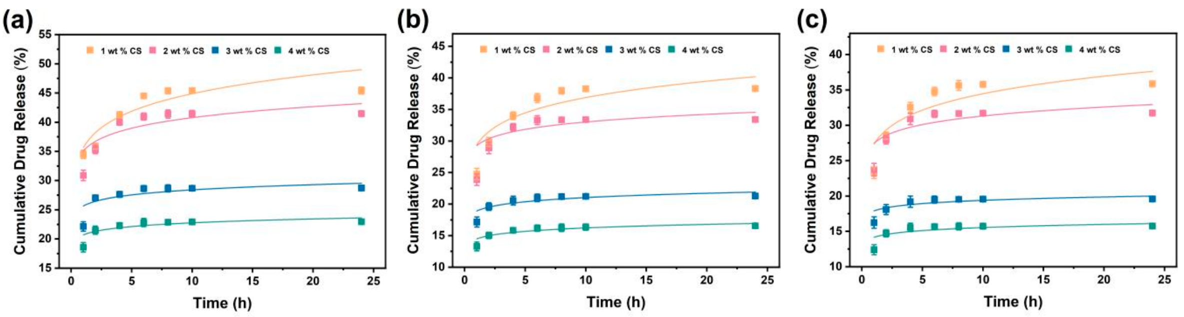

Figure S3. Ritger–Peppas model fitting of in vitro drug release profiles of A@C/P drug-loaded membranes with different CS contents under different pH conditions: (a) pH 6.8, (b) pH 7.4, and (c) pH 8.0.
